# Nitrile versus Latex for Glove Juice Sampling

**DOI:** 10.1371/journal.pone.0110686

**Published:** 2014-10-15

**Authors:** Timothy F. Landers, Anthony Dent

**Affiliations:** College of Nursing, The Ohio State University, Columbus, Ohio, United States of America; Cleveland Clinic, United States of America

## Abstract

The objective of this study was to explore the utility of nitrile gloves as a replacement for latex surgical gloves in recovering bacteria from the hands. Two types of nitrile gloves were compared to latex gloves using the parallel streak method. Streaks of *Klebsiella pneumoniae* and *Staphylococcus aureus* were made on tryptic soy agar plates, and the zones of inhibition were measured around pieces of glove material placed on the plates. Latex gloves produced a mean zone of inhibition of 0.28 mm, compared to 0.002 mm for nitrile gloves (p<.001). While the parallel streak method is not intended as a quantitative estimate of antimicrobial properties, these results suggest that nitrile may be a viable alternative to latex in glove juice sampling methods, since nitrile avoids the risk of latex exposure.

## Introduction

Hand hygiene is widely recognized as the most important means of preventing infection. Use of effective hand hygiene agents is required as part of comprehensive hand hygiene programs implemented to reduce infections in health care settings. Hand hygiene is also a critical component of occupational safety programs in high-risk settings, in the food service industry, and as a public health measure during outbreaks. With increasing concern about the burden of healthcare-associated infections and the development of antibiotic resistant organisms, improving hand hygiene by health care workers has been identified as a major patient safety initiative throughout the world [1].

Currently available hand hygiene products include soaps and alcohol-based hand rubs such as gels, lotions, foams, and liquids. Standardized methods are used to evaluate the antimicrobial properties of these products by regulatory and governmental agencies prior to approval. [2]–[5] Methods to demonstrate efficacy include using the pads of the fingers from test subjects or the recovery of bacteria using the “glove juice method” in which organisms are recovered from hands placed in oversized gloves containing sampling fluid. The sampling fluid is formulated to remove bacteria from the skin while neither promoting, nor inhibiting bacterial growth. [3] World Health Organization guidelines on hand hygiene programs recommend ongoing studies to evaluate and amend protocols to obtain valid estimates of product efficacy [1].

In glove juice protocols, specifications for reagents and sampling supplies have been developed to permit product comparison. Gloves used for sampling are generally required to be loose and un-powdered. The current ASTM International standard for surgical hand scrub preparations, ASTM Standard E1115, specifies that gloves for sampling should be “loose-fitting, unlined, powder-free latex gloves which possess no antimicrobial properties, or equivalent”. [3] Furthermore, E1115 specifies a sole source for these latex surgical gloves.

However, latex allergy represents a serious concern, particularly for health care workers. It is estimated that up to 12% of all health care workers experience a range of reactions to latex including skin irritation, local itching and burning, and allergic symptoms. [6] Death from anaphylaxis of both health care workers and patients has been reported in the literature.

Alternatives to latex gloves include those composed of nitrile, vinyl and other synthetic materials. Given the risk of latex allergy and the availability of substitute synthetic materials, non-latex alternatives would be preferred if these materials have no impact on bacterial recovery and perform well during the manipulation required for sampling.

Because latex allergy poses risk of allergic reactions, other materials would be preferred if they also meet the criteria of being easily acquired, are appropriate to the test protocol, and do not exhibit antimicrobial properties. Thus, the goal of this project was to evaluate the properties of two types of sterile nitrile gloves as an alternative to the ASTM Standard E1115-specified latex gloves.

## Materials and Methods

The antimicrobial properties of commercially available nitrile gloves were compared to those of latex gloves using the parallel streak method (AATCC 147 [7]). Briefly, five streaks of two bacterial species, *Klebsiella pneumoniae* (ATCC No. 4352) and *Staphylococcus aureus* (ATCC No. 6538), were made on plates of tryptic soy agar. A sterile sample of a glove material measuring approximately 2.5 cm×6.5 cm was then placed across the streaks.

The three types of gloves tested in the study were: 1) Acclaim Latex gloves (control), 2) KIMTECH PURE G3 Sterile STERLING Nitrile Gloves (Product no. 11828, Kimberly-Clark Professional, Roswell, GA), and 3) KIMTECH PURE G3 Sterile White Nitrile Gloves (Product no. 56887, Kimberly-Clark Professional, Roswell, GA). Different lot numbers of each test material were used, and testing of each lot was performed in triplicate. Positive controls (streaks without glove material) and negative controls (uninoculated plates) were also prepared.

After 24 hours of incubation at 35°C, the plates were examined and bacterial inhibition produced by a test material was calculated by measuring the clear zone of no growth(d) and subtracting the material width (w). Because inhibition occurs on both sides of the test material, the zone of inhibition (Z) was calculated as: Z = (w–d)/2 [7].

Mean zones of inhibition produced by each glove type were calculated and the results from nitrile gloves were compared to those of latex by a t-test of independent samples using SPSS v21 (IBM Corporation, Armonk, NY).

## Results

Three types of gloves were tested with the parallel streak method. [7] One lot of reference latex gloves and three lots of each of two types of nitrile gloves were tested for their antibacterial properties versus *Klebsiella pneumoniae* and *Staphylococcus* aureus. Three plates of each glove material were prepared for challenge by each species using five inoculum streaks on each plate.

Thus, zones of inhibition were measured for 30 streaks versus the reference material and 60 versus each test material.

**Figure 1:**
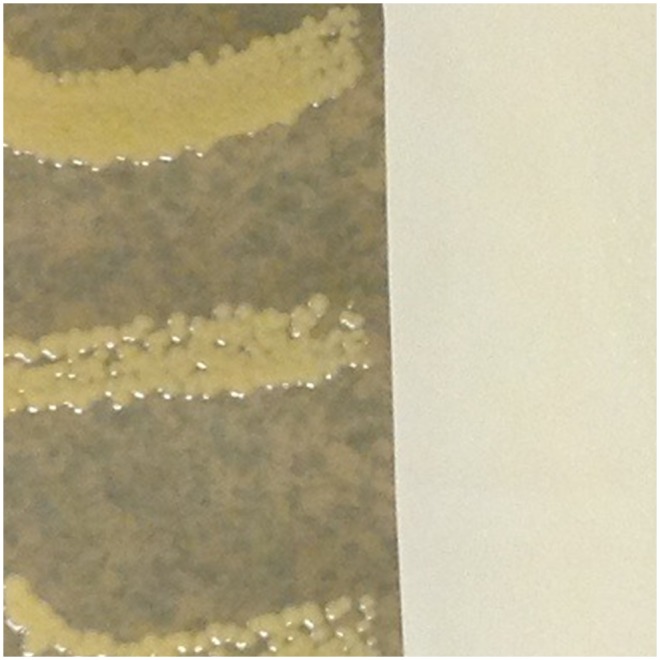
Typical results, latex gloves with inhibition of *Staphylococcus aureus* demonstrated.

**Figure 2:**
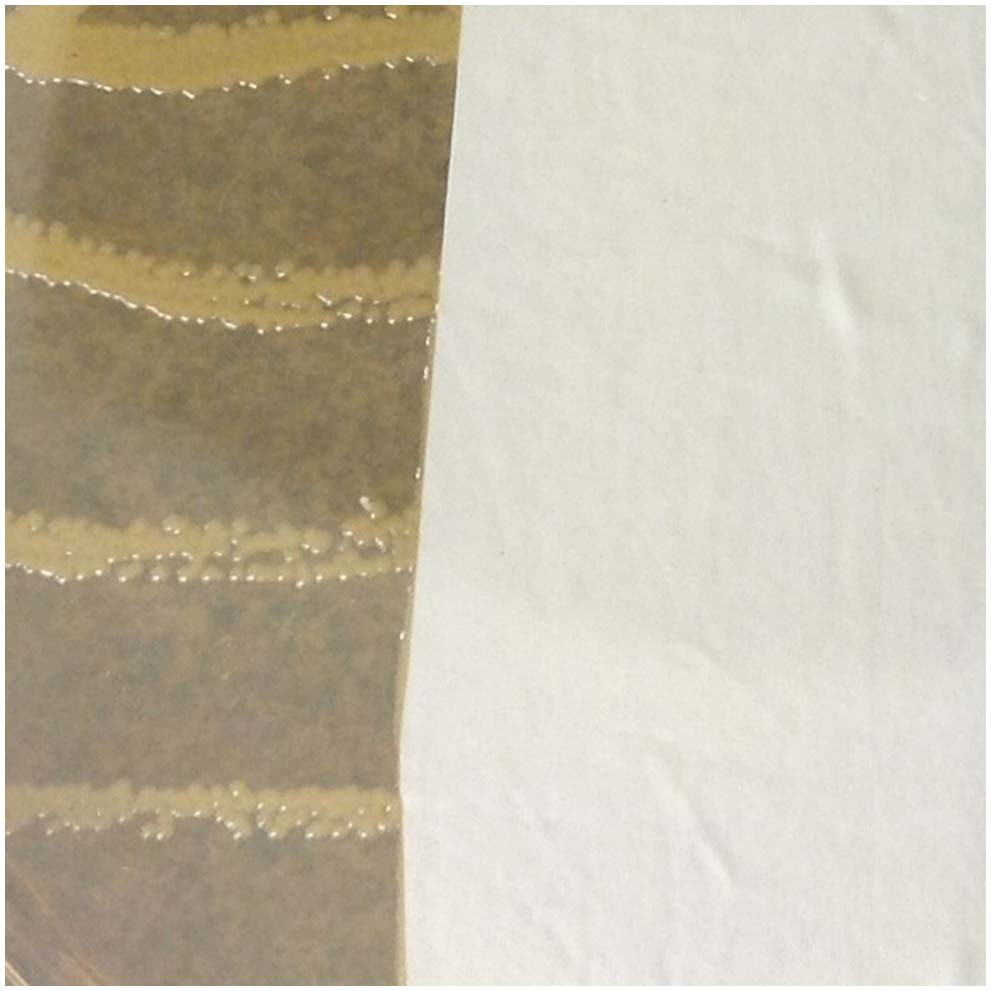
Typical results, nitrile gloves with no inhibition of *Staphylococcus aureus* demonstrated.

**Figure 3:**
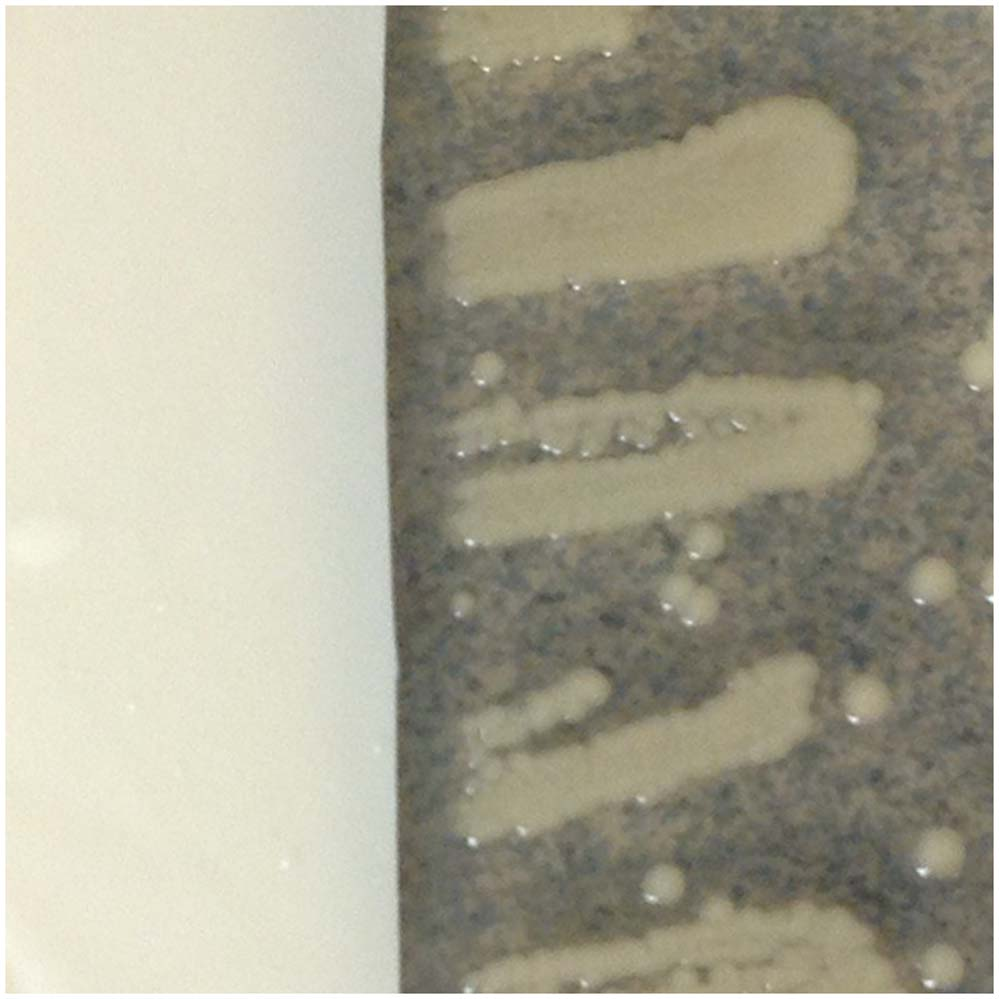
Typical results, latex gloves with inhibition of *Klebsiella pneumoniae* demonstrated.

**Figure 4:**
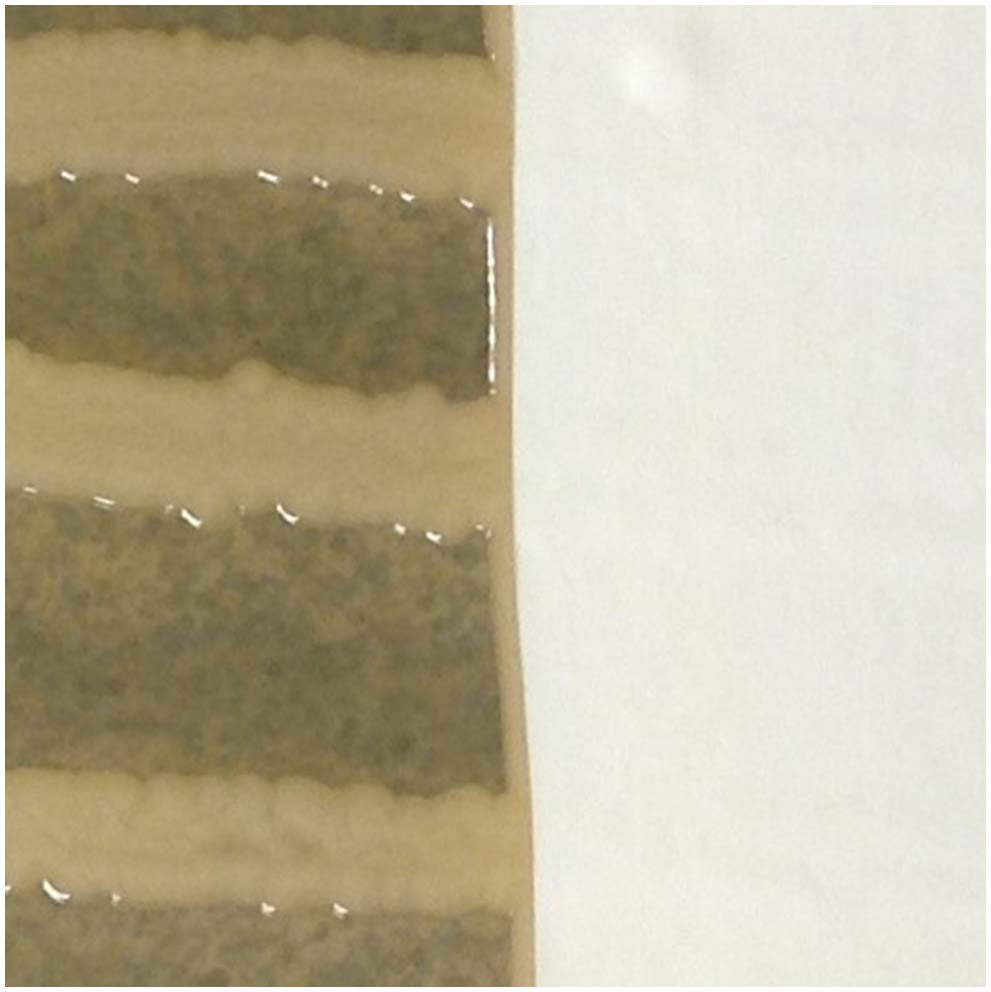
Typical results, nitrile gloves with no inhibition of *Klebsiella pneumoniae* demonstrated.

Typical results for representative glove types are shown in Figures 1–4. Overall mean zones of inhibition are shown in Table 1 and detailed results are presented in Tables S1–S6.

**Table 1:** Mean zones of inhibition (cm) among three types of glove materials.

Glove material			
	*K. pneumoniae*	*S. aureus*	Overall
Reference	0.31	0.25	0.28*
Nitrile 1	0.00	0.003	0.002*
Nitrile 2	0.00	0.00	0.00*

*p<.001.

Reference material was latex gloves, nitrile 1 was KIMTECH PURE G3 Sterile Gloves, and nitrile 2 was KIMTECH PURE G3 Sterile White Nitrile Gloves.

Both positive and negative controls were appropriate.

## Discussion

The results of this study demonstrate that two types of nitrile gloves exhibit less bacterial inhibition than do latex gloves and suggest that nitrile may be a preferred alternative to latex for use in glove juice sampling.

Although latex is a natural product derived from *Hevea brasiliensis*, it contains compounds – including Hev b antigens – capable of producing an Ig-E mediated hypersensitivity reaction in exposed and sensitized individuals. [8] Latex allergy is an important issue, especially for healthcare workers who are also targeted for most interventions to improve hand hygiene. Of note, testing procedures for hand hygiene products do not require actual intended users such as healthcare workers or food handlers; however, avoiding latex-containing products, when possible, is widely recommended. [9], [10] Strategies to prevent latex allergy include avoiding latex products when an alternative is available, prominent labelling of latex-containing products, use of low-protein, low-powder gloves, and prompt recognition and treatment of symptoms in sensitized individuals [9], [10].

Standard test methods are important in testing for comparison of products. Factors such as product volume and the relevance of contamination concentration required by current test standards should be considered when developing test protocols that reflect *in vivo* product effectiveness by end users. Standardized test protocols and research protocols currently exclude testing on individuals with known sensitivity to latex products, and this limits the applicability of current glove juice sampling methods in a proportion of real-world users [3], [11].

In addition to efficacy-testing of hand hygiene products, identifying bacteria on the hands is important to understanding variations in the microbiome and its role in initiating disease. [12], [13] Sampling the hands by swab has been shown to recover lower numbers of *Staphylococcus* spp. and *Pseudomonas* spp., but higher numbers of other bacteria, compared to glove juice sampling. [14] Thus, glove juice sampling is an important tool in understanding changes in the microbiome and its role in disease transmission.

An important limitation of this study is that it did not address the in-use impact of glove type on bacterial recovery during performance of glove juice sampling. At the 75 ml volume of sampling fluid specified by protocol, there may be little impact of latex inhibition on actual test results. Further, the flexibility of glove material is important to proper performance of glove juice sampling, but this property was not assessed in this experiment. However, in our experience, nitrile gloves are flexible enough to support the procedure.

A further limitation of this study is that only two species of bacteria were tested. Other microbial species may exhibit different susceptibility to the antimicrobial properties of glove material. In other studies, plant-derived latex has been shown to produce *in-vitro* bacterial and fungal inhibition similar to that of ciprofloxacin and fluconazole, respectively, reinforces the need for neutral materials in experimental protocols. [15] Additionally, virucidal properties of glove materials are equally relevant to standard test methods for demonstrating antiviral efficacy. [4] Further studies on the role of glove material in inhibiting bacterial growth may be required to validate these findings. The zones of inhibition, while statistically significant between latex and nitrile, were small, and the parallel streak method is not intended to be a quantitative measure of antibacterial properties.

Future research could explore performance characteristics of glove materials such as flexibility and durability, the impact of nitrile on test outcome, and bacterial adhesion.

## Conclusion

Using the parallel streak method, nitrile gloves demonstrated less bacterial inhibition than did latex gloves. Given the concern over latex allergy and the need to test hand hygiene products for antimicrobial efficacy, use of nitrile gloves offers significant advantage over latex gloves for glove juice sampling.

## Supporting Information

Table S1 Inhibition zones, reference Encore Acclaim Latex Surgical Gloves [Product #105613].
(PDF)

Table S2 Inhibition zones, Nitrile Type 1 - KIMTECH PURE G3 Sterile STERLING (Kimberly-Clark Professional, Size 10 [Product #11828, Lot #440812]).
(PDF)

Table S3 Inhibition zones, Nitrile Type 1 - KIMTECH PURE G3 Sterile STERLING (Kimberly-Clark Professional, Size 9 [Product #11827, Lot #440713]).
(PDF)

Table S4 Inhibition zones, Nitrile Type 2 - KIMTECH PURE G3 Sterile White Nitrile Gloves, Size 9 (Kimberly-Clark Professional [Product #56894, Lot #970312]).
(PDF)

Table S5 Inhibition zones, Nitrile Type 2 - KIMTECH PURE G3 White Nitrile Gloves, Size 8 (Kimberly-Clark Professional, Size 8 [Product #56892, Lot #420990]).
(PDF)

Table S6 Inhibition zones, Nitrile Type 2 - KIMTECH PURE G3 Sterile White Nitrile Gloves, Size 10 (Kimberly-Clark Professional [Product #56887, Lot #970311]).
(PDF)
