# Supplementary material for: Nitrile versus Latex for Glove Juice Sampling
Source: PLoS One. 2014 Oct 15;9(10):e110686. doi: 10.1371/journal.pone.0110686 (PMC4198239; doi:10.1371/journal.pone.0110686)
Supplement: Table S1 — Inhibition zones, reference Encore Acclaim Latex Surgical Gloves [Product #105613]. (PDF) [file pone.0110686.s001.pdf]

K. pneumonia

Plate - Streak	Test Specimen Width (mm)	Total Width (mm)	Clear zone (W) (mm)
1-1	2.6	2.9	0.15
1-2	2.6	3	0.2
1-3	2.7	3	0.15
1-4	2.7	3	0.15
1-5	2.7	2.9	0.1
2-1	2.1	2.3	0.1
2-2	2.2	2.4	0.1
2-3	2.1	2.3	0.1
2-4	2.1	2.4	0.15
2-5	2.1	2.5	0.2
3-1	1.9	2.1	0.1
3-2	1.7	2.1	0.2
3-3	1.7	2.1	0.2
3-4	1.8	2.2	0.2
3-5	1.8	2.2	0.2
**Mean**			**0.31**
**SD**			**0.04**

S. aureus

Plate - Streak	Test Specimen Width (mm)	Total Width (mm)	Clear zone (W) (mm)
1-1	2.6	2.8	0.1
1-2	2.6	2.8	0.1
1-3	2.6	2.8	0.1
1-4	2.6	2.9	0.15
1-5	2.6	2.8	0.1
2-1	2	2.2	0.1
2-2	2.1	2.4	0.15
2-3	2.1	2.5	0.2
2-4	2.2	2.5	0.15
2-5	2.2	2.6	0.2
3-1	2.2	2.3	0.05
3-2	2.1	2.4	0.15
3-3	2.1	2.3	0.1
3-4	2	2.2	0.1
3-5	2	2.3	0.15
**Mean**			**0.25**
**SD**			**0.04**

Overall Mean (mm) **0.28**

.
