# Supplementary material for: Nitrile versus Latex for Glove Juice Sampling
Source: PLoS One. 2014 Oct 15;9(10):e110686. doi: 10.1371/journal.pone.0110686 (PMC4198239; doi:10.1371/journal.pone.0110686)
Supplement: Table S5 — Inhibition zones, Nitrile Type 2 - KIMTECH PURE G3 White Nitrile Gloves, Size 8 (Kimberly-Clark Professional, Size 8 [Product #56892, Lot #420990]). (PDF) [file pone.0110686.s005.pdf]

K. pneumoniae

Plate - Streak	Test Specimen Width (mm)	Total Width (mm)	Clear zone (W) (mm)
1-1	2	2	0
1-2	2	2	0
1-3	2	2	0
1-4	2	2	0
1-5	2	2	0
2-1	2.4	2.4	0
2-2	2.4	2.4	0
2-3	2.4	2.4	0
2-4	2.5	2.5	0
2-5	2.4	2.4	0
3-1	2.3	2.3	0
3-2	2.1	2.1	0
3-3	2	2	0
3-4	1.9	1.9	0
3-5	1.9	1.9	0
**Mean**			**0**

S. aureus

Plate - Streak	Test Specimen Width (mm)	Total Width (mm)	Clear zone (W) (mm)
1-1	2.1	2.1	0
1-2	2.1	2.1	0
1-3	2.1	2.1	0
1-4	2.1	2.1	0
1-5	2.1	2.1	0
2-1	2.3	2.3	0
2-2	2.4	2.4	0
2-3	2.4	2.4	0
2-4	2.5	2.5	0
2-5	2.4	2.4	0
3-1	2.1	2.1	0
3-2	2	2	0
3-3	2	2	0
3-4	2	2	0
3-5	2	2	0
**Mean**			**0**

Overall Mean (mm) **0**

.
