# Supplementary material for: Nitrile versus Latex for Glove Juice Sampling
Source: PLoS One. 2014 Oct 15;9(10):e110686. doi: 10.1371/journal.pone.0110686 (PMC4198239; doi:10.1371/journal.pone.0110686)
Supplement: Table S6 — Inhibition zones, Nitrile Type 2 - KIMTECH PURE G3 Sterile White Nitrile Gloves, Size 10 (Kimberly-Clark Professional [Product #56887, Lot #970311]). (PDF) [file pone.0110686.s006.pdf]

K. pneumoniae

Plate - Streak	Test Specimen Width (mm)	Total Width (mm)	Clear zone (W) (mm)
1-1	2	2	0
1-2	2	2	0
1-3	1.9	1.9	0
1-4	1.7	1.7	0
1-5	1.9	1.9	0
2-1	1.6	1.6	0
2-2	1.7	1.7	0
2-3	1.7	1.7	0
2-4	1.7	1.7	0
2-5	1.7	1.7	0
3-1	1.7	1.7	0
3-2	1.7	1.7	0
3-3	1.7	1.7	0
3-4	1.7	1.7	0
3-5	1.7	1.7	0
**Mean**			**0**

S. aureus

Plate - Streak	Test Specimen Width (mm)	Total Width (mm)	Clear zone (W) (mm)
1-1	1.7	1.7	0
1-2	1.8	1.8	0
1-3	1.8	1.8	0
1-4	1.8	1.8	0
1-5	1.8	1.8	0
2-1	1.6	1.6	0
2-2	1.7	1.7	0
2-3	1.7	1.7	0
2-4	1.7	1.7	0
2-5	1.7	1.7	0
3-1	1.7	1.7	0
3-2	1.7	1.7	0
3-3	1.7	1.7	0
3-4	1.7	1.7	0
3-5	1.7	1.7	0
**Mean**			**0**

Overall Mean (mm) **0**
